# MRI-measured adipose features as predictive factors for detection of prostate cancer in males undergoing systematic prostate biopsy: a retrospective study based on a Chinese population

**DOI:** 10.1080/21623945.2022.2148885

**Published:** 2022-11-24

**Authors:** Tianyu Xiong, Fang Cao, Guangyi Zhu, Xiaobo Ye, Yun Cui, Huibo Zhang, Yinong Niu

**Affiliations:** a Department of Urology, Beijing Chaoyang Hospital, Capital Medical University, Beijing, China; b Department of Urology, Beijing Shijitan Hospital, Capital Medical University, Beijing, China; c Department of Radiology, Beijing Chaoyang Hospital, Capital Medical University, Beijing, China

**Keywords:** Adipose tissue, prostate cancer, nomogram, magnetic resonance imaging, biopsy

## Abstract

In this study, we retrospectively evaluated the data of 901 men undergoing ultrasonography-guided systematic prostate biopsy between March 2013 and May 2022. Adipose features, including periprostatic adipose tissue (PPAT) thickness and subcutaneous fat thickness, were measured using MRI before biopsy. Prediction models of all PCa and clinically significant PCa (csPCa) (Gleason score higher than 6) were established based on variables selected by multivariate logistic regression and prediction nomograms were constructed. Patients with PCa had higher PPAT thickness (4.64 [3.65–5.86] vs. 3.54 [2.49–4.51] mm, *p* < 0.001) and subcutaneous fat thickness (29.19 [23.05–35.95] vs. 27.90 [21.43–33.93] mm, *p* = 0.013) than those without PCa. Patients with csPCa had higher PPAT thickness (4.78 [3.80–5.88] vs. 4.52 [3.80–5.63] mm, *p* = 0.041) than those with non-csPCa. Adding adipose features to the prediction models significantly increased the area under the receiver operating characteristics curve for the prediction of all PCa (0.850 *vs*. 0.819, *p* < 0.001) and csPCa (0.827 *vs*. 0.798, *p* < 0.001). Based on MRI-measured adipose features and clinical parameters, we established two nomograms that were simple to use and could improve patient selection for prostate biopsy in Chinese population.

## Introduction

The incidence of prostate cancer (PCa) in China has increased noticeably in recent years [1,2]. The active development of prostate-specific antigen (PSA) screening and improvements in magnetic resonance imaging (MRI) are important factors underlying this increase in PCa detection [3]. However, there are still a considerable number of negative biopsy cases among men with elevated PSA levels or suspected lesions on MRI. Excess prostate puncture biopsies not only increase the risk of complications, such as bleeding, haematuria, infection, and urinary retention, but also impose economic and psychological burdens on patients [4–6]. Furthermore, the potential risk of overdiagnosis and overtreatment of early PCa should be taken into account [7].

Several previous studies have reported nomograms for predicting the occurrence of PCa on biopsy, but their clinical value is limited because of the difficulty in obtaining the biomarkers proposed in these prediction models, such as urinary prostate cancer antigen 3 and the Prostate Health Index [8–12]. Two nomograms were developed by the European Randomized Study of Screening for Prostate Cancer and Memorial Sloan Kettering Cancer Center to improve patient selection for prostate biopsy [13,14]. However, these predictive tools were established based on databases of patients from Europe and North America, which may limit their application in the Chinese population. In addition, patients with bone metastasis or obvious extracapsular lesions that should have been detected by MRI before biopsy were analysed together with patients with early cancer, resulting in potential confounding factors. Therefore, the clinical value and accuracy of these predictive models could be improved by adding new clinical characteristics that are easy to obtain. In addition, it is necessary to build a nomogram based on a database of Chinese PCa patients to generate more accurate prediction models for the Chinese population.

The relationship between obesity and PCa is a significant but not wholly understood topic [15–18]. Previous studies have suggested that adipose tissue distribution is associated with PCa progression [19]. Periprostatic adipose tissue (PPAT), the metabolically active fat tissue surrounding the prostate gland, has recently emerged as a potential factor in PCa development [20]. The adipose tissue anterior to the prostate gland and posterior to the symphysis pubis, called the ‘prostatic anterior fat pad’ in some manuscripts, is significantly correlated with PCa aggressiveness [21–23]. Some researchers have quantified the thickness of this pre-prostatic adipose pad using ultrasonography, computed tomography, and MRI [19,24–27]. PPAT thickness is significantly correlated with higher Gleason scores, advanced tumour stage, and poor prognosis after treatment, reflecting a trend of more aggressive cancer [19,24–27]. However, studies on the predictive value of PPAT thickness for PCa on biopsy are limited. Further, there are few studies on the relationship between imaging features of subcutaneous fat tissue and PCa development. Some have reported that a high subcutaneous fat mass is associated with better survival outcomes in patients with PCa, but the relationship between subcutaneous fat and PCa detection in prostate biopsy remains unclear [28,29].

The aim of the current study was to analyse the predictive value of MRI-measured PPAT thickness and subcutaneous fat tissue for all PCa and clinically significant PCa (csPCa) among Chinese men who undergoing systematic biopsy. We also built predictive nomograms based on the clinical parameters and adipose features to improve patient selection for prostate biopsy.

## Materials and methods

### Patient selection and data collection

In this retrospective study, we analysed the data of patients who underwent transrectal ultrasonography-guided systematic prostate biopsy between March 2013 and May 2022 at the Beijing Chaoyang Hospital because of elevated PSA levels (>4.0 ng/mL), MRI-visible lesions, or suspicious nodules on digital rectal examination. All prostate biopsies were performed with at least 10 systematic cores under transrectal ultrasonography guidance by experienced urologists. The highest Gleason score among all biopsy cores was documented as the biopsy Gleason score. csPCa was defined as PCa with a Gleason score of 3 + 4 or greater. Non-csPCa was defined as PCa with a total Gleason score ≤6. The cohort was divided into cancer and non-cancer groups based on the pathological biopsy results. The cancer group was further divided into csPCa and non-csPCa groups based on Gleason scores. Pathological results and clinical parameters, including age, body mass index (BMI), and the latest serum PSA levels prior to biopsy, were collected retrospectively by reviewing medical records. If a patient underwent repeated biopsies, only the results from the last biopsy were included in the database. The following exclusion criteria were used: (1) incomplete data, (2) malignant disease not originating from the prostate, (3) obvious extracellular extension, seminal vesicle invasion, rectal invasion, or bone metastatic lesions indicated by MRI, and (4) non-Asian ethnicity.

Our study was conducted in accordance with the Declaration of Helsinki (as revised in 2013) and was approved by the Institutional Review Board of Beijing Chaoyang Hospital, Capital Medical University (NO.: 2022-Ke-55), which waived the requirement of informed consent for this retrospective analysis. This study was adhered to the Transparent Reporting of a Multivariable Prediction Model for Individual Prognosis or Diagnosis (TRIPOD) statement on reporting prediction models [30].

### Image analysis

All patients were routinely evaluated using multiparametric prostate MRI prior to biopsy. MRI was performed using a 3.0-Tesla scanner with a pelvic phased-array surface coil without an endorectal coil. All magnetic resonance images were retrospectively reviewed by a radiologist with more than a decade of experience in reviewing prostate magnetic resonance images. Lesions on MRI were assigned a Prostate Imaging Reporting and Data System (PI-RADS) score of 1 to 5 based on the PI-RADS version 2.1 guideline [31]. The prostate volume was calculated using the traditional prolate ellipse formula, where volume = (width × height × length) × π/6 [32]. All prostate dimensions were measured on MRI scans.

**Figure 1.**
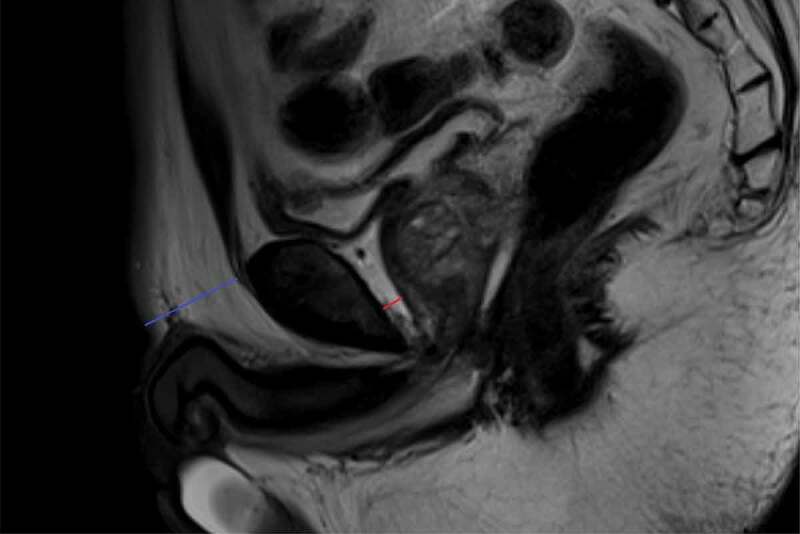
Measurement of PPAT thickness (marked red) and subcutaneous fat thickness (marked blue). PPAT: periprostatic adipose tissue.

Adipose features were measured manually using the Picture Archiving and Communication System (Centricity PACS Version 6.0 SP, GE Healthcare, Chicago, USA) in our hospital. PPAT thickness was measured as the shortest perpendicular distance from the pubic symphysis to the prostate on a single T2-weighted midsagittal plane (marked in red in Figure 1). Similarly, subcutaneous fat thickness was measured as the shortest perpendicular distance from the pubic symphysis to the skin on the same slice where PPAT thickness was measured (marked in blue in Figure 1). All measurement procedures were performed by two urologists with at least 5-year experience who knew that the patients had undergone prostate biopsy but were blinded to the clinical and pathological information. The average of the results measured by the two urologists was used in the statistical analysis. The inter-observer reliability was tested using intraclass correlation coefficient (ICC).

### Statistical analysis and nomogram establishment

Continuous variables are expressed as medians with interquartile ranges and were compared using the Mann-Whitney test. To facilitate the analysis and model design, categorical variables, including age (<70 years *vs*. ≥70 years), BMI (<25 kg/m^2^ *vs*. ≥25 kg/m^2^), PI-RADS score (PI-RADS 1–3 *vs*. PI-RADS 4–5), prostate volume (<40 cm^3^ *vs*. ≥40 cm^3^), PSA level (≤10 ng/mL *vs*. 10–20 ng/mL *vs*. ≥20 ng/mL), PPAT thickness (≤3 mm *vs*. 3–4 mm *vs*. 4–5 mm *vs*. 5–6 mm *vs*. ≥6 mm), and subcutaneous fat thickness (<30 mm *vs*. ≥30 mm), were converted to categorical variables and compared using the chi-square test or Fisher’s exact test, as appropriate.

To establish the prediction models, covariate candidates were analysed using logistic regression analysis. Variables with a *p*-value <0.1 in the univariate analyses were further assessed using multivariate analyses. Prediction models were established based on variables with a *p*-value <0.05 in the multivariate logistic regression analyses. To test the discriminative ability of each model, receiver operating characteristic (ROC) curve analysis was performed, and the area under the curve (AUC) and 95% confidence interval (CI) were calculated.

To test the additional predictive power of PPAT and subcutaneous fat thicknesses, we also built prediction models based only on clinical parameters. The DeLong test was used to compare the AUCs between the models with and without adipose features. We also evaluated the degree of improvement of adding adipose features to the prediction models by calculating the net reclassification improvement (NRI), which is the sum of the gain in both sensitivity and specificity for a given risk threshold. For each model, decision curve analysis was performed to further analyse the net benefits of the prediction models. The net benefit of our study can be interpreted as the benefit of omitting unnecessary prostate biopsy in healthy men with results suggestive of PCa on PSA screening or MRI scans, subtracting the harm of delayed detection and subsequent treatment for PCa patients.

Based on the multivariable logistic regression models with adipose features, two composite nomograms were constructed for all PCa and csPCa. Robustness assessment of the two models was performed by internal validation using bootstrap resampling. Bootstrap samples were randomly drawn with replacements from the original sample. The models were repeatedly fitted to 1000 bootstrap samples, and the AUC was computed for each iteration. The mean AUC from the bootstrap samples was then calculated.

Calibration of the models was assessed by comparison of the predicted and observed risks of all PCa and csPCa. Calibration curves were constructed for both models, showing the correlation between the predicted probability versus the observed positive rate in deciles of patients with increasing values of the predicted probability. Plots were drawn by a loess smoother algorithm to allow more insight into calibration analysis. The goodness of fit of the model was tested using the Hosmer-Lemeshow test.

SPSS version 26.0 (IBM Corp., Armonk, NY, USA) and R version 4.1.2 (http://www.r-project.org/) were utilized for statistical analysis. All statistical tests were two-tailed, and *p* < 0.05 was considered significant for all parameters.

## Results

### Patient characteristics

**Figure 2.**
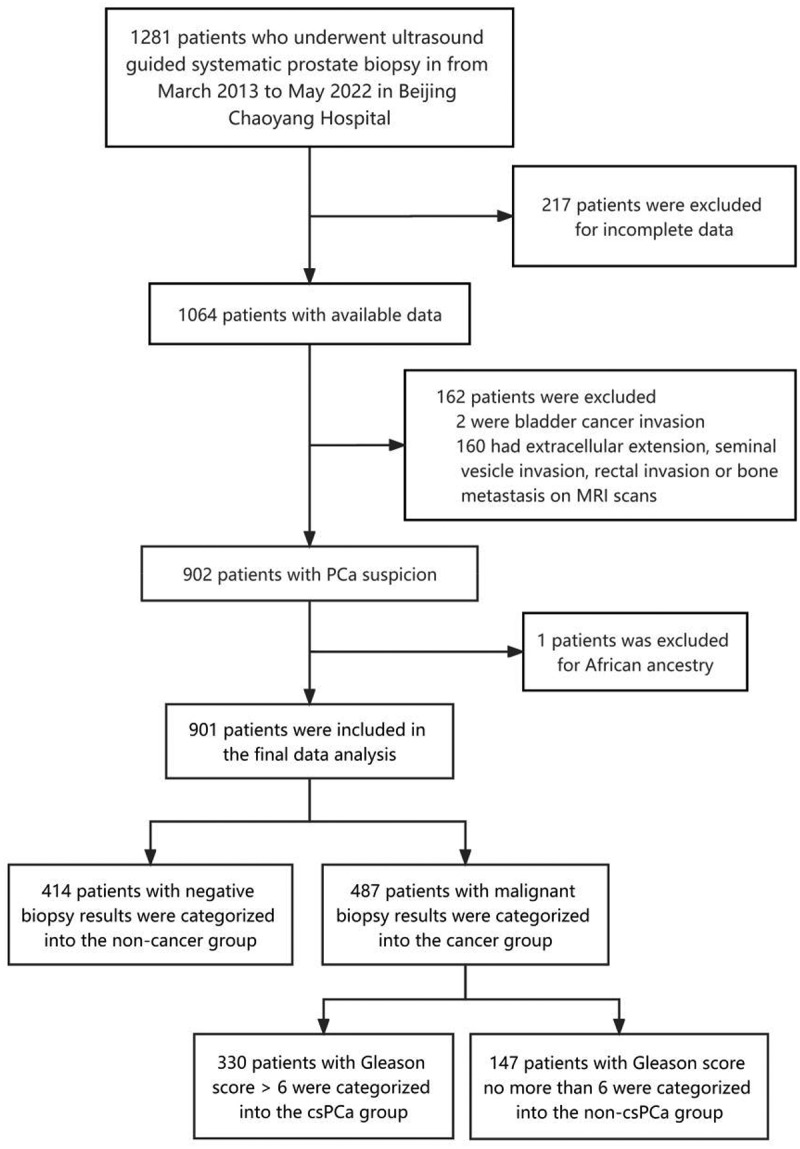
Patient selection and exclusion. PCa: prostate cancer; csPCa: clinically significant prostate cancer.

A total of 1281 consecutive patients who underwent ultrasonography-guided systematic prostate biopsy were assessed for inclusion in the present study. A total of 380 patients were excluded according to the exclusion criteria (Figure 2), resulting in a cohort of 901 patients. A total of 414 men with negative biopsy results, such as benign prostate hyperplasia or prostatitis, were included in the non-cancer group, and 487 men with malignant biopsy results were included in the cancer group, which was further divided into the csPCa group (Gleason score >6, n = 340) and the non-csPCa group (Gleason score ≤6, n = 147).

The characteristics and adipose tissue measurement results of all patients were presented in Table 1. The median age and BMI were 68 years and 24.79 kg/m^2^, respectively. A total of 538 (59.7%) men had a PI-RADS score of 4 or 5. The median PSA was 12.11 ng/mL, and 345 (38.3%) patients had a PSA level less than 10 ng/mL. The median PPAT and subcutaneous fat thicknesses were 4.11 mm and 28.65 mm, respectively. Excellent reproducibility of measurement was determined using ICC and assessed as 0.964 for PPAT (*p* < 0.001) and 0.975 for subcutaneous fat thicknesses (*p* < 0.001).

**Table 1.** Clinical characteristics and adipose features of patients by pathological results of systematic prostate biopsy.

	Overall				Cancer patients		
Variables	Total (n = 901)	Non-cancer group (n = 414)	Cancer group (n = 487)	*p*-value^a^	Non-csPCa group (n = 147)	csPCa group (n = 340)	*p*-value^b^
Age (year), median (IRQ)	68 (63–75)	65 (60–71)	70 (65–76)	**<0.001**	70 (65–76)	70 (65–76)	0.387
Age group, n (%)				**<0.001**			0.948
< 70 years	519 (57.6)	286 (69.1)	233 (47.8)		70 (47.6)	163 (47.9)	
≥ 70 years	382 (42.4)	128 (30.9)	254 (52.2)		77 (52.4)	177 (52.1)	
BMI (kg/m^2^), median (IRQ)	24.79 (22.66–26.69)	24.51 (22.29–26.69)	24.80 (22.86–26.70)	0.176	24.74 (22.86–26.56)	24.91 (22.86–26.83)	0.576
BMI group, n (%)				0.376			0.688
< 25 kg/m^2^	484	229	255		79 (53.7)	176 (51.8)	
≥ 25 kg/m^2^	417	185	232		68 (46.3)	164 (48.2)	
PI-RADS score, n (%)				**<0.001**			**0.006**
PI-RADS 1–3	363 (40.3)	270 (65.2)	93 (19.1)		39 (26.5)	54 (15.9)	
PI-RADS 4–5	538 (59.7)	144 (34.8)	394 (80.9)		108 (73.5)	286 (84.1)	
Prostate volume (cm^3^), median (IRQ)	40.63 (29.15–59.96)	50.99 (33.75–73.16)	34.01 (25.60–40.10)	**<0.001**	36.40 (28.83–52.11)	33.14 (25.02–44.82)	**0.016**
Prostate volume group, n (%)				**<0.001**			
< 40 cm^3^	441 (48.9)	140 (33.8)	301 (61.8)		86 (58.5)	215 (63.2)	0.324
≥ 40 cm^3^	460 (51.1)	274 (66.2)	186 (38.2)		61 (41.5)	125 (36.8)	
PSA (ng/mL), median (IRQ)	12.11 (7.98–23.46)	10.06 (7.17–16.60)	15.11 (9.16–30.13)	**<0.001**	10.14 (7.08–16.06)	18.62 (11.00–36.52)	**<0.001**
PSA group, n (%)				**<0.001**			**<0.001**
≤ 10 ng/mL	345 (38.3)	200 (48.3)	145 (29.8)		69 (46.9)	76 (22.3)	
10–20 ng/mL	291 (32.3)	129 (31.2)	162 (33.3)		53 (36.1)	109 (32.1)	
≥ 20 ng/mL	265 (29.4)	85 (20.5)	180 (36.9)		25 (17.0)	155 (45.6)	
PPAT thickness (mm), median (IRQ)	4.11 (2.96–5.31)	3.54 (2.49–4.51)	4.64 (3.65–5.86)	**<0.001**	4.52 (3.80–5.63)	4.78 (3.80–5.88)	**0.041**
PPAT thickness group, n (%)				**<0.001**			0.406
≤ 3 mm	229 (25.4)	155 (37.4)	74 (15.2)		27 (18.4)	47 (13.8)	
3–4 mm	193 (21.4)	106 (25.6)	87 (17.9)		31 (21.1)	56 (16.5)	
4–5 mm	201 (22.3)	81 (19.6)	120 (24.6)		35 (23.8)	85 (25.0)	
5–6 mm	144 (16.0)	48 (11.6)	96 (19.7)		26 (17.7)	70 (20.6)	
≥ 6 mm	134 (14.9)	24 (5.8)	110 (22.6)		28 (19.0)	82 (24.1)	
Subcutaneous fat thickness (mm), median (IRQ)	28.65 (22.14–34.72)	27.90 (21.43–33.93)	29.19 (23.05–35.95)	**0.013**	28.65 (22.36–34.37)	29.48 (23.25–36.20)	0.307
Subcutaneous fat thickness group, n (%)				**0.007**			0.360
< 30 mm	505 (56.0)	252 (60.9)	253 (52.0)		81 (55.1)	172 (50.6)	
≥ 30 mm	396 (44.9)	162 (39.1)	234 (48.0)		66 (44.9)	168 (49.4)	

*p* < 0.05 is indicated by boldface.

^a^The *p*-values were compared between cancer group and non-cancer group. ^b^ The *p*-values were compared between non-csPCa group and csPCa group.

IQR: interquartile range; csPCa: clinically significant prostate cancer; BMI: body mass index; PI-RADS: Prostate Imaging Reporting and Data System; PSA: prostate-specific antigen; PPAT: periprostatic adipose tissue.

Compared to those in the non-cancer group, patients in the cancer group were older, had higher PSA levels, and were more likely to have a PI-RADS score of 4–5, but had a smaller prostate volume (all *p*-values <0.001). There was no significant difference between the two groups in BMI (*p* = 0.176). The PPAT thickness (4.64 [3.65–5.86] mm *vs*. 3.54 [2.49–4.51] mm, *p* < 0.001) and subcutaneous fat thickness (29.19 [23.05–35.95] mm *vs*. 27.90 [21.43–33.93] mm, *p* = 0.013) were significantly higher in the cancer group than in the non-cancer group. Among patients with PCa, those in the csPCa group had higher PSA levels, a higher proportion of patients with PI-RADS scores of 4–5, a smaller prostate volume (all *p*-values <0.001) than those in the non-csPCa group. Patients with csPCa also had higher PPAT thickness than those without csPCa (4.78 [3.80–5.88] mm *vs*. 4.52 [3.80–5.63] mm, *p* = 0.041).

### Nomogram development

**Figure 3.**
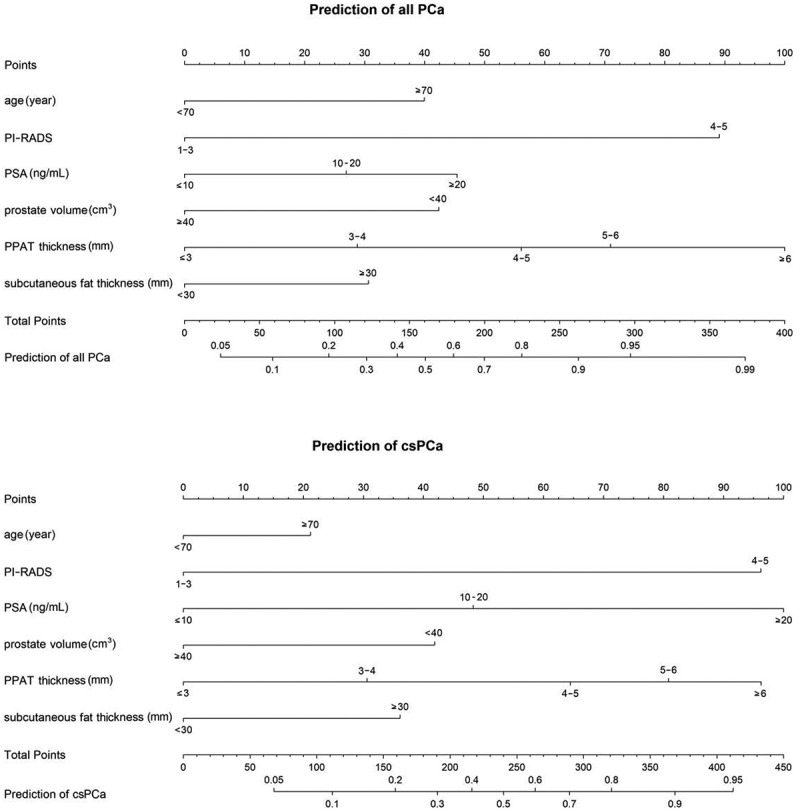
Nomograms for prediction of all PCa and csPCa. Predictors included clinical parameters and adipose features. PCa: prostate cancer; csPCa: clinically significant prostate cancer; PI-RADS: Prostate Imaging Reporting and Data System; PSA: prostate-specific antigen; PPAT: periprostatic adipose tissue.

Among the variables evaluated, univariate logistic regression analyses showed that age, PI-RADS score, prostate volume, PSA, PPAT thickness, and subcutaneous fat thickness were significantly correlated with the detection of all PCa and csPCa in systematic prostate biopsy (Table 2). Multivariate analysis showed that these variables were all independent predictors of PCa and csPCa (all *p*-values <0.05). Prediction models were then built, and two composite nomograms were constructed (Figure 3).

**Table 2.** Univariate and multivariate logistic regression analyses for prediction of all PCa and csPCa.

Variables	Prediction of all PCa				Prediction of csPCa			
	Univariate analysis		Multivariate analysis		Univariate analysis		Multivariate analysis	
	OR (95% CI)	*p-*value	OR (95% CI)	*p-*value	OR (95% CI)	*p*-value	OR (95% CI)	*p*-value
Age ≥ 70 years	2.436 (1.852–3.203)	**<0.001**	2.360 (1.671–3.334)	**<0.001**	1.886 (1.435–2.479)	**<0.001**	1.434 (1.030–1.995)	**0.033**
BMI ≥ 25 kg/m^2^	1.126 (0.866–1.465)	0.376			1.134 (0.866–1.486)	0.360		
PI-RADS score 4–5	7.944 (5.866–10.758)	**<0.001**	6.856 (4.871–9.649)	**<0.001**	6.494 (4.645–9.079)	**<0.001**	5.205 (3.602–7.523)	**<0.001**
Prostate volume < 40 cm^3^	0.316 (0.240–0.415)	**<0.001**	0.400 (0.282–0.568)	**<0.001**	0.392 (0.297–0.518)	**<0.001**	0.488 (0.345–0.691)	**<0.001**
PSA group								
≤ 10 ng/mL	-	-	-	-	-	-	-	-
10–20 ng/mL	1.732 (1.264–2.373)	**0.001**	1.788 (1.207–2.648)	**0.004**	2.120 (1.497–3.002)	**<0.001**	2.289 (1.536–3.411)	**<0.001**
≥ 20 ng/mL	2.921 (2.089–4.083)	**<0.001**	2.682 (1.758–4.090)	**<0.001**	4.987 (3.504–7.098)	**<0.001**	5.577 (3.656–8.447)	**<0.001**
PPAT thickness group								
≤ 3 mm	-	-	-	-	-	-	-	-
3–4 mm	1.719 (1.157–2.555)	**0.007**	1.845 (1.144–2.975)	**0.012**	1.583 (1.157–2.555)	**0.044**	1.686 (1.008–2.821)	**0.047**
4–5 mm	3.103 (2.090–4.607)	**<0.001**	3.310 (2.023–5.414)	**<0.001**	2.837 (1.854–4.343)	**< 0.001**	2.994 (1.806–4.963)	**<0.001**
5–6 mm	4.189 (2.688–6.528)	**<0.001**	4.513 (2.601–7.832)	**<0.001**	3.663 (2.317–5.790)	**<0.001**	3.950 (2.265–6.889)	**<0.001**
≥ 6 mm	9.600 (5.701–16.168)	**<0.001**	8.474 (4.561–15.744)	**<0.001**	6.106 (3.806–9.798)	**<0.001**	5.158 (2.927–9.089)	**<0.001**
Subcutaneous fat thickness ≥ 30 mm	1.274 (0.978–1.660)	**0.073**	1.796 (1.276–2.528)	**0.001**	1.343 (1.024–1.761)	**0.033**	1.785 (1.277–2.495)	**0.001**

*p* < 0.1 in the univariate analysis and *p* < 0.05 in the multivariate analysis are indicated by boldface.

OR: odds ratio; CI: confidence interval; PCa: prostate cancer; csPCa: clinically significant prostate cancer; BMI: body mass index; PSA: prostate-specific antigen; PI-RADS: Prostate Imaging Reporting and Data System; PPAT: periprostatic adipose tissue.

**Figure 4.**
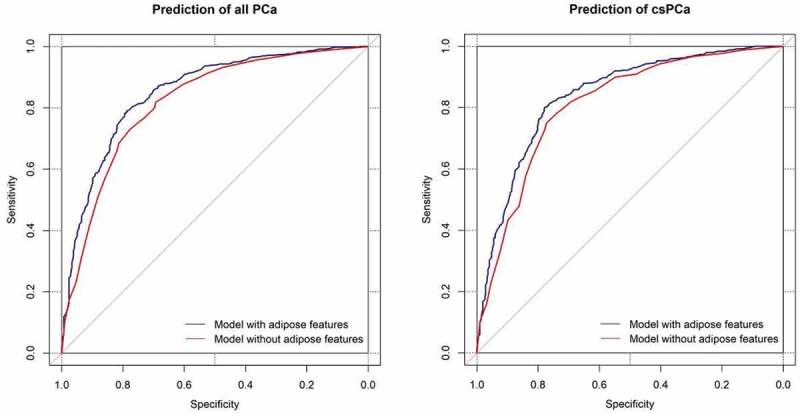
ROC curves of prediction models with or without adipose features. Models with adipose features had significantly higher AUC for prediction of all PCa [AUC = 0.850 (95% CI 0.825–0.876) *vs*. 0.819 (0.791–0.847), *p* < 0.001] and prediction of csPCa [AUC = 0.827 (95% CI 0.801–0.854) *vs*. 0.798 (0.769–0.827), *p* < 0.001]. Adipose features included periprostatic adipose tissue thickness and subcutaneous fat thickness. ROC: receiver-operating-characteristic; AUC: area under curve; PCa: prostate cancer; csPCa: clinically significant prostate cancer.

**Figure 5.**
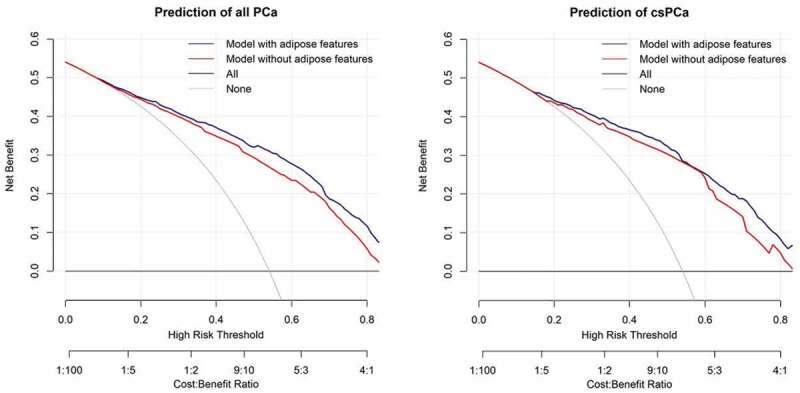
Decision curve analysis for models with or without adipose features predicting all PCa and csPCa in systematic prostate biopsy. Models with adipose features had higher net benefit for the entire range of risk thresholds. Adipose features included periprostatic adipose tissue thickness and subcutaneous fat thickness. PCa: prostate cancer; csPCa: clinically significant prostate cancer.

The AUCs of the prediction models were 0.850 (95% CI 0.825–0.876) for all PCa and 0.819 (95% CI 0.801–0.854) for csPCa, which were significantly higher than those of the models without adipose features (Figure 4). Improved predictive power by the addition of adipose features was also evidenced by overall categorical NRIs of 0.276 (95% CI 0.139–0.420) for all PCa and 0.165 (95% CI 0.102–0.337) for csPCa using a 10% risk difference as the cut-off (both *p* < 0.001). An assessment of the total NRI along with the changes in sensitivity and specificity is provided in the Supplement table 1 and 2. We further applied decision curve analysis, as shown in Figure 5. The models with adipose features had a higher net benefit for the entire range of risk thresholds. Internal validation of the MIA nomogram using bootstrap techniques (1000 repetitions) resulted in a mean AUC of 0.778 for predicting all PCa and 0.748 for csPCa.

**Figure 6.**
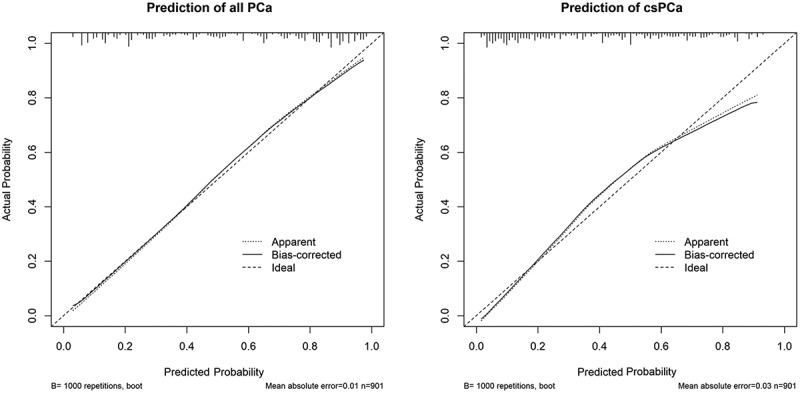
Local regression nonparametric smoothing plot showing the calibration of the nomograms for all PCa and csPCa. The ideal line estimated probabilities corresponding to the actual observed. The apparent line represented prediction capability of the model obtained after data analysis. The bias-corrected line showed prediction capability of the model obtained after bootstrap correction. Vertical lines at the top of the figure represent number of patients. PCa: prostate cancer; csPCa: clinically significant prostate cancer.

The calibration plots (Figure 6) indicated that the nomograms were well calibrated for both PCa and csPCa. The *p*-values for the Hosmer-Lemeshow test of the two models were 0.613 and 0.192, respectively, indicating good agreement between observed and predicted probabilities.

## Discussion

In recent years, PPAT has emerged as a potential factor promoting the development of PCa [20]. Although it is located in the pelvic region, PPAT is often considered visceral adipose tissue and has vigorous metabolic activity [20]. It is becoming increasingly clear that PPAT is biologically distinct from other adipose tissues. Unlike other adipose depots, elevated levels of chronic inflammatory and fibrosis factors have been observed in PPAT, and its volume is independent of the state of obesity [33,34]. A range of mechanisms have been proposed to explain the effects of PPAT on PCa development, including the release of growth factors, stimulation of inflammatory signalling activators, hyperinsulinemia, altered adipokine profiles, and increased lipid availability [35–37].

Several previous studies have evaluated the association between the imaging features of PPAT and PCa aggressiveness. In 2010, van Roermund *et al*. first reported a correlation between PPAT and high-risk PCa in patients undergoing radiotherapy [25]. They measured the cross-sectional area of the PPAT on a single transverse section and created a normalized index called PPAT density by dividing the PPAT area by the total pelvic area. In subsequent studies, various standards and measurement methods for PPAT were adopted. PPAT thickness, measured as the shortest perpendicular distance between the pubic bone and prostate, is the simplest and most widely used PPAT imaging feature. This parameter is correlated with higher Gleason scores, a shorter interval to castration-resistant prostate cancer, lower overall survival after androgen deprivation therapy, and upstaging from stage cT1/2 to pT3 in radical prostatectomy [19,24–27]. These studies have demonstrated that PPAT thickness is associated with adverse clinical and prognostic features in patients with PCa. Only one previous study has investigated the PPAT features in individuals without a prior PCa diagnosis. Bhindi *et al*. reported that PPAT thickness measured by transrectal ultrasonography was a predictor of PCa and high-grade PCa at biopsy [24]. However, the measurement of PPAT thickness using ultrasonography is affected by the subjective judgement of operators, which is a potential source of measurement bias.

In the present study, we analysed the predictive value of MRI-measured PPAT thickness for PCa in prostate biopsy. We found that PCa patients had a significantly higher PPAT thickness than healthy men, and, similarly, patients with csPCa (Gleason score >6) had a higher PPAT thickness than those with non-csPCa (Gleason score ≤6). Logistic regression analyses showed that PPAT thickness was an independent predictor of both PCa and csPCa. Our results are in accordance with those of a previous study and provide further evidence that a higher PPAT thickness is a positive predictive indicator of PCa [24].

The association between obesity and poor treatment outcomes of PCa has been discussed in several studies; however, this relationship remains inconclusive [15–18]. In our study, there was no significant difference in BMI between the cancer and non-cancer groups. In contrast, PCa patients had a higher subcutaneous fat thickness than healthy men. These contradicting results may be because the BMI, which is used to define obesity, provides no insight into the distribution of adipose tissue. Adipose tissue distributed near the prostate gland, including abdominal subcutaneous fat, probably plays a more important role in PCa development than other adipose depots.

To further explore the predictive value of PPAT and subcutaneous fat thicknesses, we built prediction models based on variables selected by multiple logistic regression analyses. The addition of adipose features significantly increased the AUCs of the models for all PCa (0.850 *vs*. 0.819, *p* < 0.001) and csPCa (0.827 *vs*. 0.798, *p* < 0.001), with overall categorical NRIs of 0.276 and 0.165, respectively. The decision curve analysis showed that models with adipose features had higher net benefits for the entire range of risk thresholds. These results indicate that our new nomograms accurately predict the risk of PCa in prostate biopsies. The internal validation results demonstrated the good accuracy of the two nomograms (AUC = 0.778 and 0.748, respectively).

In our study, we excluded patients with extracapsular extension, seminal vesicle invasion, rectal invasion, or bone metastasis detected before biopsy. These imaging features are strong predictors of PCa and correlate with a very high cancer incidence [38,39]. Therefore, we believe that the baseline conditions of patients with these characteristics could not match those of other patients, especially for prediction models aimed at men with suspicion of PCa. These patients were excluded to avoid the effects of potential confounding factors on the prediction models. The ethnicity, as a probable confounding factor, has also been reported to influence both PCa risk and PPAT character [40]. Therefore, we excluded one patient with African ancestry from the study cohort.

When converting the clinical and adipose parameters into categorical variables, we chose thresholds based on clinical significance and convenience of application. BMI > 25 kg/m^2^, age ≥70 years old and prostate volume ≥40 cm^3^ are often used to distinguish overweight, elder patients and large prostates [17,20,21,41,42]; patients with PI-RADS score 4 have high risk of cancer and clinically significant cancer is likely to be present [31]; PSA levels of 10 and 20 ng/mL are cut-offs for risk differentiation in European association of urology (EAU) guideline [43]. For the thickness of PPAT and subcutaneous fat, convenience of clinical application is the main concern, since the measurements of adipose thickness on MRI images are easily affected by the observers. Besides, there are only few previous studies on the predictive value of adipose thickness for PCa in biopsy. As a result, we set intervals of 1 mm for PPAT thickness and cut-off of subcutaneous fat thickness ≥30 mm to minimize the influence of measurement error. Although the conversion to categorical variables sacrificed some accuracy, it increased the overall practicality of the prediction models and reduced the effect of errors caused by adipose feature measurements.

We noticed a relatively high percentage (59.7%) of PI-RADS scores 4 and 5 in our study, which can be explained by the fact that the males in our study were not from a cohort subjected to prostate cancer screening. Instead, they underwent biopsy because of abnormal PSA level, MRI scan or digital rectal examination. Thus, it seems reasonable to expect more of PI-RADS scores 4/5 cases among them.

Our study had some limitations. First, it was a retrospective, single-institution study, resulting in potential selection bias. Further, data of rectal examination and family history could not be retrieved and external validation could not be performed. Second, it has been reported that the PPAT level might be influenced by the long-term use of 5-α reductase inhibitors (5-ARIs) [44]. We could not retrieve the medical history of 5-ARI use in the patients, and this potential drug-induced change was not analysed in our study. Conversely, we noticed that a small prostate volume, which was possibly caused by 5-ARI use, was correlated with the risks of PCa and csPCa in our study. Therefore, further studies are warranted to explore the effect of 5-ARI use on PPAT changes and PCa risk. Third, our results were based on transrectal ultrasonography-guided systematic biopsy, which may not be applicable to other approaches and techniques. Fourth, the biopsy Gleason scores may have been underestimated. Because some non-csPCa patients in our series did not receive surgical treatment, we did not evaluate the upgrading rate of Gleason scores in radical prostatectomy.

In conclusion, we analysed the adipose features measured by MRI scans in a cohort of Chinese patients undergoing systematic biopsy for suspected PCa. We found that PPAT thickness and subcutaneous fat thickness were both significantly associated with the detection of PCa and csPCa in prostate biopsy. Our nomograms are simple to use and can refine patient selection for prostate biopsy.

## Supplementary Material

Supplemental Material

## Data Availability

The data that support the findings of this study are available on request from the corresponding author, Yinong Niu. The data are not publicly available due to restriction by the Institutional Review Board of Beijing Chaoyang Hospital, Capital Medical University, in order to protect the patient privacy.
