# Supplementary material for: MRI-measured adipose features as predictive factors for detection of prostate cancer in males undergoing systematic prostate biopsy: a retrospective study based on a Chinese population
Source: Adipocyte. 2022 Nov 24;11(1):653–64. doi: 10.1080/21623945.2022.2148885 (PMC9704414; doi:10.1080/21623945.2022.2148885)
Supplement: Supplemental Material [file KADI_A_2148885_SM1566.docx]

Supplementary Table 1 Comparison of the models with or without adipose features at various cut-offs for the probability of all prostate cancer in biopsy. Comparative outcomes are sensitivity gain, specificity gain, and total NRI. NRI: net reclassification index.

| **Probability cut-off** | **Sensitivity gain (%)** | **Specificity gain (%)** | **Total NRI** |
| --- | --- | --- | --- |
| 5% | 19.9 | 28.5 | 0.484 |
| 6% | 14.7 | 24.4 | 0.391 |
| 7% | 14.6 | 20.5 | 0.351 |
| 8% | 14.6 | 20.7 | 0.353 |
| 9% | 12.7 | 19.5 | 0.322 |
| 10% | 11.7 | 15.9 | 0.276 |
| 11% | 9.7 | 11.3 | 0.210 |
| 12% | 8.4 | 10.6 | 0.190 |
| 13% | 6.6 | 8.9 | 0.155 |
| 14% | 5.7 | 8.0 | 0.137 |
| 15% | 4.9 | 8.7 | 0.136 |

Supplementary Table 2 Comparison of the models with or without adipose features at various cut-offs for the probability of clinically significant prostate cancer in biopsy. Comparative outcomes are sensitivity gain, specificity gain, and total NRI. NRI: net reclassification index.

| **Probability cut-off** | **Sensitivity gain (%)** | **Specificity gain (%)** | **Total NRI** |
| --- | --- | --- | --- |
| 5% | 20.9 | 15.7 | 0.366 |
| 6% | 21.8 | 11.0 | 0.328 |
| 7% | 21.8 | 8.9 | 0.307 |
| 8% | 17.6 | 6.8 | 0.244 |
| 9% | 15.0 | 6.4 | 0.214 |
| 10% | 9.4 | 7.1 | 0.165 |
| 11% | 11.1 | 5.5 | 0.161 |
| 12% | 11.2 | 4.1 | 0.153 |
| 13% | 10.3 | 5.0 | 0.153 |
| 14% | 11.5 | 4.4 | 0.159 |
| 15% | 8.5 | 3.9 | 0.124 |
